# Competition for Trophies Triggers Male Generosity

**DOI:** 10.1371/journal.pone.0018050

**Published:** 2011-04-06

**Authors:** Xiaofei Sophia Pan, Daniel Houser

**Affiliations:** Interdisciplinary Center for Economic Science, George Mason University, Fairfax, Virginia, United States of America; University of Zürich, Switzerland

## Abstract

**Background:**

Cooperation is indispensable in human societies, and much progress has been made towards understanding human pro-social decisions. Formal incentives, such as punishment, are suggested as potential effective approaches despite the fact that punishment can crowd out intrinsic motives for cooperation and detrimentally impact efficiency. At the same time, evolutionary biologists have long recognized that cooperation, especially food sharing, is typically efficiently organized in groups living on wild foods, even absent formal economic incentives. Despite its evident importance, the source of this voluntary compliance remains largely uninformed. Drawing on costly signaling theory, and in light of the widely established competitive nature of males, we hypothesize that unique and displayable rewards (trophies) out of competition may trigger male generosity in competitive social environments.

**Principal Findings:**

Here, we use a controlled laboratory experiment to show that cooperation is sustained in a generosity competition with trophy rewards, but breaks down in the same environment with equally valuable but non-unique and non-displayable rewards. Further, we find that males' competition for trophies is the driving force behind treatment differences. In contrast, it appears that female competitiveness is not modulated by trophy rewards.

**Significance:**

Our results suggest new approaches to promoting cooperation in human groups that, unlike punishment mechanisms, do not sacrifice efficiency. This could have important implications in any domain where voluntary compliance matters — including relations between spouses, employers and employees, market transactions, and conformity to legal standards.

## Introduction

Promoting the behaviour of altruists and depressing that of egoists is vital for cooperation [1], [2]. The axiom of self-interested behaviour suggests that to accomplish this, one must provide sufficient individual incentives [3], [4]. Pecuniary incentives are natural, but in enforcing compliance they can sacrifice intrinsic altruistic motives and reduce economic efficiency [2], [5]–[14].

Status, a non-pecuniary reward that is potentially efficient, can also be an effective incentive. For example, evolutionary biologists have long pointed to status as the reason that human males in hunter-gatherer societies provide food to their group even absent direct food reciprocity [15]–[19]. In particular, such contributions are displays that generate status for the winners of hunting competitions [19]. In this sense, status emerges as a currency of reciprocity [11]–[13], [20].

Given that displays can lead to status, and that status improves males reproductive success [21], it follows that males may compete for unique displays out of competition [15],[16],[18],[22]. If so, an intrinsic desire for unique and displayable rewards out of competition might impact male behaviour in contemporary competitive environments. This effect could perhaps be used to promote generosity efficiently in social environments.

We examined how a competition for unique and displayable (trophy) rewards affects male generosity in a ‘public goods’ game with real money stakes. A total of 152 subjects (30.8% female) participated in our experiment under three conditions: the *Mug* treatment (a unique and displayable mug); the *Ice-cream* treatment (Haagen-Dazs ice-cream bar rewards [see picture S1]) and the *Baseline* (absent rewards).

**Figure 1:**
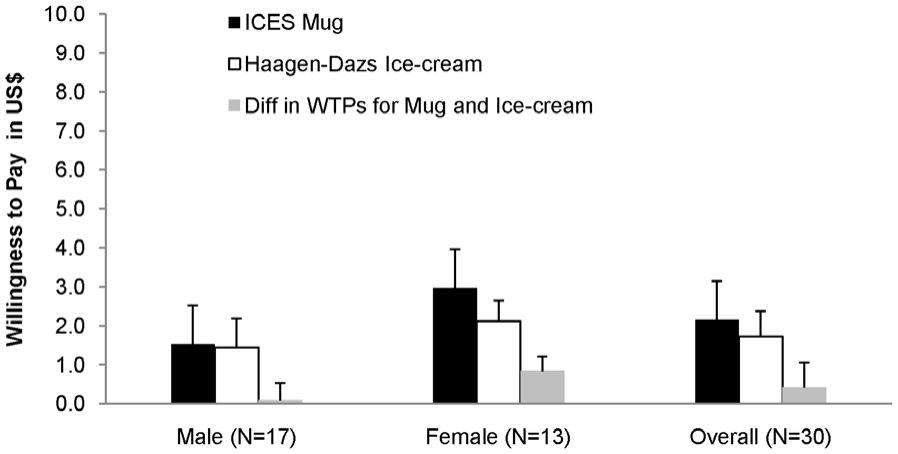
Willingness-to-pay (WTP) for ICES Mug and Haagen-Dazs Ice-cream. The Fig. describes males' and females' WTP for the ICES Mug (filled black bars), the Haagen-Dazs Ice-cream (open bars), and the differences between them (filled grey bars). WTPs are statistically identical between males and females for both the mug (z = 1.593, P = 0.111) and the Ice-cream bar (z = 1.418, P = 0.156). WTPs are also statistically identical within the same gender for the two items (Wilcoxson signed-rank test: for male, z = 0.049, P = 0.961; for female, z = 0.956, P = 0.339, two-tailed). The differences in WTP for Mug and Ice-cream are also statistically identical between males and females (z = 0.727, P = 0.467).

Given that systematic differences in the way males and females value the mug and ice-cream rewards could have confounded inferences regarding sources of behaviours among treatments, we addressed this by conducting a standard random-auction Willingness-to-pay (henceforth WTP) elicitation [23] where subjects received identical information about ice-cream and mug as those in the ‘public goods’ game (see Methods). We were unable to find evidence of systematic differences in subjective values between either males and females or mug and ice-cream (Fig. 1; unless otherwise noted, all p-values are based on two-tailed Mann-Whitney tests).

The *Ice-cream* condition constitutes a powerful control for the effects of trophies on participants' decisions. In particular, *Ice-cream* is identical to *Mug* except that the Ice-cream reward is neither unique nor displayable. Comparing *Mug* to *Ice-cream* thus provides rigorous evidence on how competition for displayable rewards influences cooperation. Moreover, given that subjects know that rewards will be distributed privately, our design enables an investigation of how rewards modulate subjects' intrinsic desire to compete [24], [25] (see Methods Summary).

All interactions in the experiment took place anonymously. In all conditions, fixed groups of four subjects played a game they knew would last ten periods. Each group member received an endowment of 20 Experimental Dollars (henceforth E$, 1 US $ = 25 E$), and decided how much to contribute to a group project. All E$s not contributed to the project were transferred to the subject's private account. For every 1E$ contributed to the project, all group members, including those who invested little or nothing, earned 0.4 E$. Thus, in the *Baseline* treatment, it was always in a participant's material self-interest to invest 0 E$, regardless of the contributions of the participant's group members. In the reward treatments, one had an added incentive to contribute, but our WTP elicitation (Fig. 1) suggests these incentives are small and identical between reward treatments (see SI: Text S1). Note that if every group member chose to keep her or his endowment privately, then there was nothing to be shared, whereas if all contributed their entire 20 E$ then every member earned 0.4×80 = 32 E$.

Subjects made their contribution decisions simultaneously. Afterwards, they were shown the contribution decisions of each of their (anonymous and non-gender identified) group members. Decisions were displayed in a random order to avoid reputation building (see methods summary). Then, subjects were able to assign approval ratings to each of their group members. All approval decisions were made simultaneously and subjects were not able to assign approval to themselves. At the end of each period in the *Mug* and *Ice-cream* conditions, subjects who received the most approval points won an electronic gold star. In case of ties, all earned a gold star, so that each subject could receive up to ten stars over ten periods. Each star increased the chance to win the final reward by 10 percentage points. Thus, a person with zero gold stars at the end of the game had a zero percent chance to win the award, while a person with 10 gold stars won the award with probability one.

## Results and Discussion

**Figure 2:**
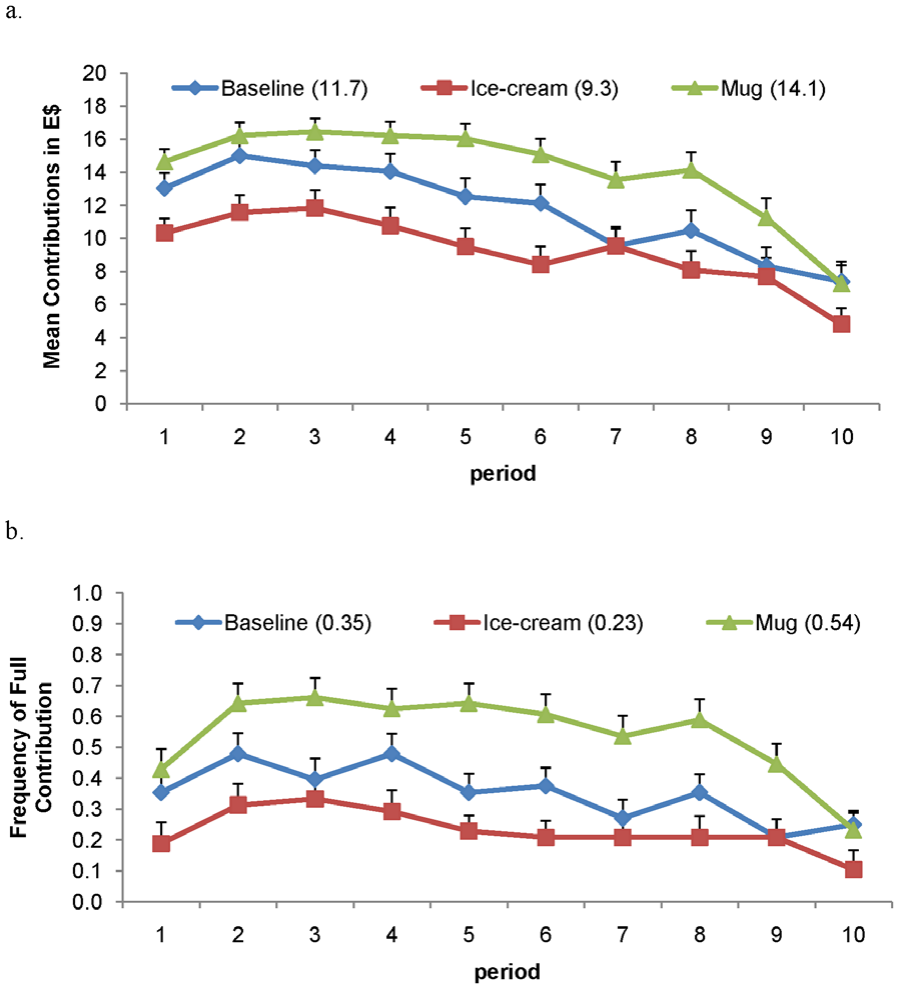
Contributions to the public goods over 10 periods across treatments. Cooperation is highest in Mug both by a) average contribution or b) frequency of the full contribution. a. The numbers in parentheses indicate mean contribution (over 10 periods) for that treatment. Contributions are significantly higher in *Mug* (N = 14 groups) compared to both *Ice-cream* (N = 12 groups, z = 2.675, P = 0.008) and *Baseline* (N = 12 groups, z = −1.800, P = 0.072). b. The numbers in parentheses indicate mean frequency (over 10 periods) of full contributions in that treatment. In the *Mug* treatment, most subjects contributed their full endowment (54%), significantly more than in both *Baseline* (35%, N = 12 groups, z = −1.987, P = 0.047) and *Ice-cream* (23%, z = 2.734, P = 0.006).

Higher contributions relative to group members led to higher approval in all treatments (see SI: Table S1). Despite this similarity, we find that overall contributions are significantly higher in *Mug* than in either *Ice-cream* or *Baseline* (Fig. 2a). Moreover, the frequency of full contributions is highest in *Mug*. For example, from period 6 to 10, 48.2% of subjects in *Mug* contributed their full endowment, while only 29.2% did so in *Baseline* and 18.8% in *Ice-cream* (Fig. 2b).

**Figure 3:**
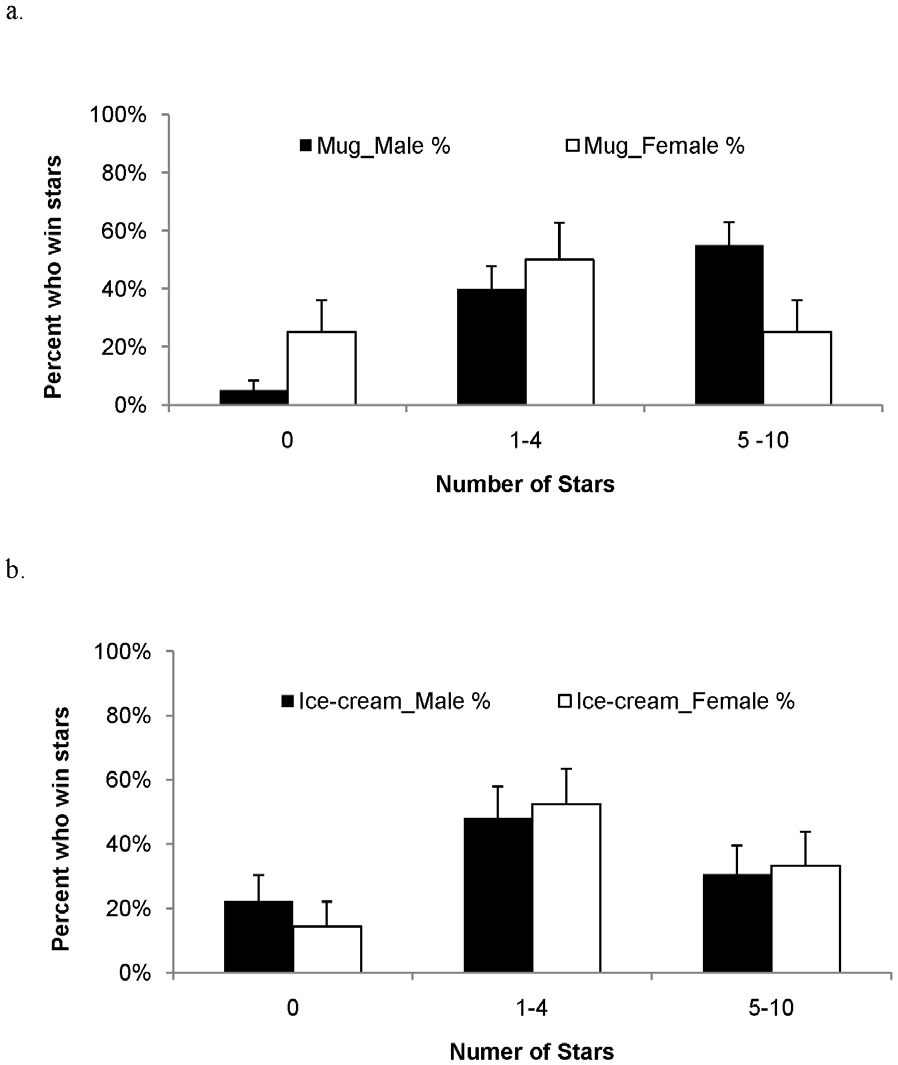
Number of stars won in *Mug* and *Ice-cream* treatments. Each panel describes the percent of males and females who won different numbers of stars (tying allowed) in *Mug* or *Ice-cream*. a. Percent of males (filled bars, N = 40) and females (open bars, N = 16) winning 0, 1–4 or 5–10 stars in *Mug*. Significantly more males than females won at least one star, or at least five stars over 10 periods. b. Percent of males (filled bars, N = 27) and females (open bars, N = 21) winning 0, 1–4, or 5–10 stars in *Ice-cream*.

Higher cooperation in *Mug* is associated with increased male competitiveness in relation to Ice-cream. Significantly more males in *Mug* (N = 40) won at least one star over ten periods than did males in *Ice-cream* (N = 27, z = 2.116, P = 0.034), while females display no difference between *Mug* (N = 16) and *Ice-cream* (N = 21) (z = −0.813, P = 0.16). Also, males are significantly more competitive than females in *Mug*. More males (95%) than females (75%) won at least one star (Fig. 3a, z = 2.166, P = 0.030) in *Mug*. Also, more males (55%) than females (25%) won at least five stars (z = −2.015, P = 0.044). In contrast, we find no evidence of differences between males (N = 27) and females (N = 21) in *Ice-cream*, either for those who have won at least one star (z = 0.692, P = 0.489), or those who won five stars or more (z = 0.000, P = 1.000, see Fig. 3b).

These gender differences, as well as male competitive generosity in *Mug*, are supported by convergent evidence from a random (individual) effect GLS regression analysis (with robust standard errors clustered by group, see, SI: Table S2). We examined how the contribution of subject *i* in the current period varied with: 1) *i'*s gender; 2) the approval points *i* received in the previous period; 3) the average contribution of *i*'s group members; and 4) period dummies. The coefficient of male in *Mug* is 5.657 (z = 2.76, P = 0.006), and the coefficient of female in *Mug* is 1.369 (z = 0.62, P = 0.536). The coefficient of *Mug* is significantly higher than the coefficient of female in *Mug*, (chi2 (1) = 4.41, P = 0.036), the coefficient of male in *Baseline*, 0.943 (chi2 (1) = 3.78, P = 0.052), and the coefficient of male in *Ice-cream*, 1.175 (chi2 (1) = 2.95, P = 0.086). This indicates, after controlling for other factors, that males in *Mug* voluntarily contribute significantly more than males or females in all other treatments and our results are robust to controlling for session effects. A random effect Tobit analysis yields substantively identical results (see, SI: Table S2).

### Discussion

What mechanism underlies the effect of trophies on male generosity? One possibility is that trophies modulate male beliefs regarding the probability of seemingly altruistic acts [1] by others. In particular, males might have expected other males to contribute more in *Mug* to compete for the mug reward. Indeed, we find higher first-period contributions by males in *Mug* than in *Ice-cream* (N = 40 and 27, respectively; z = 3.696, P = 0.000). This is not true for females (N = 16 and 21, respectively; z = 1.376, P = 0.169).

We also considered whether the effect of trophy rewards varies with one's cooperative propensity. In particular, we classified each subject as either a co-operator or free-rider based on her or his contribution in relation to group members (see SI: Text S2). The frequency of male co-operators in *Mug* (72.5%) is significantly higher than in *Ice-cream* (44.4%, z = 2.294, P = 0.022, Table 1), and similar to *Baseline* (75%, P = 0.812, z = 0.239). Nevertheless, co-operators' contributions in *Mug* (mean = 16.3, N = 12 groups) are significantly higher than in *Baseline* (mean = 13.3, N = 11 groups, P = 0.021, z = −2.309).

**Table 1:** Percent of Cooperators.

Pairwise Comparison between Male			Pairwise Comparison between Female		
Mug(N = 40)	Baseline (N = 32)	Ice-cream (N = 27)	Mug (N = 16)	Baseline (N = 16)	Ice-cream (N = 21)
73%	71%	------------	75%	63%	------------
73%**	-------------	44%	75%	-------------	71%
----------	71%**	44%	----------	63%	71%

Level of significance for Table 1–3: *p<0.1, **p<0.05, ***p<0.01.

Numbers of males/females are in parentheses.

The frequency of female co-operators is statistically identical between treatments (see Table 1). Also, female co-operators' contributions do not statistically differ between *Mug* (mean = 15.5, N = 7 groups) and *Baseline* (Table 2, mean = 14.7, N = 8 groups, z = 0.926, P = 0.354). Female co-operators' contributions are significantly higher in *Mug* than in *Ice-cream* (mean = 11.7, N = 9 groups, z = 1.747, P = 0.081). Females were not typically star-winners in *Mu*g. This is consistent with the theory that higher female contributions in *Mug* are due to males' initial unconditional generosity combined with subsequent female cooperation (see SI: Table S2).

**Table 2 pone-0018050-t002:** Mean Contribution of Cooperators.

Pairwise Comparison between Male			Pairwise Comparison between Female		
Mug (N = 12)	Baseline (N = 11)	Ice-cream (N = 10)	Mug (N = 7)	Baseline (N = 8)	Ice-cream (N = 9)
16.3**	13.3	---------------	15.5	13.7	---------------
16.3	------------	12.8	15.5*	------------	11.7
------------	13.3	12.8	---------------	13.7	11.7

Numbers in parentheses are at group level (see Text S3).

While free-riders' contributions also increase under trophy rewards (Table 3), they nevertheless remain low. It is perhaps surprising that the frequency of full contribution remains high in *Mug* through the eighth period , in light of systematic low-contributors and substantial theoretical and empirical evidence that free-riding is contagious [1],[2], [26]–[28]. One explanation is that receiving approval in *Mug* diminishes co-operators' negative emotions [29]. In particular, free-riders can reciprocate by assigning approval points to co-operators, thereby increasing the chance that a co-operator will receive the trophy reward. In view of the arguments noted above, we might expect to observe more approval assigned in *Mug* than *Ice-cream* or *Baseline*. We might also expect female free-riders to be especially generous with approval.

**Table 3:** Mean Contribution of Free-riders.

Pairwise Comparison between Male			Pairwise Comparison between Female		
Mug (N = 9)	Baseline (N = 7)	Ice-cream (N = 10)	Mug (N = 4)	Baseline (N = 4)	Ice-cream (N = 4)
8.7	7.7	---------------	7.7	6.6	---------------
8.7*	---------------	4.4	7.7*	--------------	5.2
--------------	7.7**	4.4	---------------	6.6	5.2

Numbers in parentheses are at group level (see Text S3).

Although sample sizes are small, we find that female free-riders in *Mug* (N = 4 groups, see SI: Text S3) assigned significantly more approval than either male free-riders in *Ice-cream* (N = 4, z = 2.021, P = 0.043) or female free-riders in *Ice-cream* (N = 10 groups, P = 0.048, z = 1.980). Their approval assignment was not significantly different from male free-riders in *Mug* (N = 9 groups, P = 0.217, z = 1.234) Moreover, trophy rewards do not modulate co-operators' approval decisions. Approval points assigned by female co-operators in *Mug* (N = 7) differ neither from female co-operators in *Ice-cream* (N = 9, z = 0.053, P = 0.958) nor male co-operators in *Ice-cream* (N = 10, z = 0.781, P = 0.435) or *Mug* (N = 12, z = 0.423, p = 0.673). A random effect GLS regression analysis provides additional evidence that only the approval behaviour of female free-riders is modulated by trophy rewards (see SI: Table S3).

Our results support the view that unique and displayable rewards promote male generosity and cooperation in a social dilemma environment through a generosity competition. We examined behaviour under both Ice-cream and Mug (trophy) rewards, and found only trophy rewards to promote cooperation. Our *Ice-cream* treatment rules out competition per se [30] as an explanation for increased cooperation, as it is identical to *Mug* except that ice-cream reward is neither unique nor displayable. Further, our WTP comparison between *Ice-cream* and *Mug* rules out explanations for our results that appeal to differences in subjective values males and females assign to the rewards. We speculated that the mechanism underlying cooperation with trophy rewards relies on the combination of two forces: 1) changes in expectations (especially male expectations) due to the presence of a unique and displayable reward; and 2) the use of approval by free-riders (especially female free-riders) as a currency of reciprocity.

Our results suggest new directions for designing institutions to promote cooperation efficiently among groups of genetic strangers, mechanisms that turn on reward rather than sanctions.

## Methods

A total of 182 students from George Mason University participated in our experiments. 152 subjects (34.9% female) participated in the ‘public goods’ experiments and an additional 30 subjects (43.3% female) who had not participated in the ‘public goods’ experiment took part in the hand-run WTP elicitation [23] (see methods).

A total of thirteen sessions, each with 8–12 subjects, took place for three different conditions in the ‘public goods’ experiment. Each subject only participated in one session for one condition. The experiments lasted 45–50 min and on average subjects earned $16.00 per session.

In both the *Mug* and *Ice-cream* treatment, rewards were briefly shown by the experimenter to all the subjects together in the room. In each period, the subjects knew nothing about the history of contributions of specific group members, thus ruling out reputation formation. At the end of each period in the reward treatments, subjects were informed of: 1) the accumulated gold stars they had earned; 2) the total approval they received; 3) the highest contribution among gold star winners in their group (if tied); 4) their own contribution; and 5) their current and accumulated monetary pay-off. At the end of each period in *Baseline*, subjects only know 2), 4) and 5). The experimenter distributed the reward (see methods), along with the cash payment, to each subject privately.

Upon entering the laboratory each subject, was seated in a carrel separated from other subjects in a way that ensured anonymity. Participants then received written instructions. After the experimenter read the instructions aloud, participants were quizzed to ensure they understood the procedures and the payoff structure. The experiment did not proceed until each subject completed the quiz successfully. The ‘public goods’ game was written using the experimental software Z-tree [31].

### How to distribute the rewards

Those who earned stars in *Mug* and *Ice-cream* treatments had the opportunity to draw once from a deck of ten cards, numbered 1 through 10. Subjects would receive the reward if the number they drew was equal to or smaller than the number of stars they earned during the experiment.

### Willingness-to-Pay (WTP) Elicitation

We recruited 30 students (43.3% female) who had not participated in the ‘public goods’ experiment to take part in the WTP elicitation. This experiment adopted the Becker-DeGroot-Marschak [23] random auction mechanism to elicit WTP for the ICES mug and the Haagen-Dazs ice-cream bar. Subjects were endowed with $10. Prices of the auctioned items ranged from $0 to $10 in increments of $0.50. The maximum value $10 exceeded their maximum expected WTP and the minimum $0 was at least equal to their WTP. Subjects in the WTP experiment were provided with the same information about the auctioned items as subjects in the respective rewards treatments of the ‘public goods’ game.

### Ethics Statement

All procedures used in the research are in accordance with the Guidelines for the Use of Human Subjects in Research and have been approved by the Institutional Review Board of George Mason University (HSRB protocol #6300). We obtained written consent form from all participants in our study.

## Supporting Information

Table S1 **Determinants of Approval Received.** Table S1 shows that determinants of approval points received follow a similar pattern across the treatments. In particular, the greater (smaller) the contribution in relation to others, the greater (smaller) was the amount of approval a person received. The strength of this effect is identical among treatments. This is shown by the coefficient for “Treatment variable (Baseline/Mug/Ice-cream)×Positive/Negative Deviation from Others' average.” Moreover, in all treatments, the group's highest contributor is also a star winner with frequency at least 90%.
(GIF)

Table S2 **Female Conditional Cooperation and Male Unconditional Generosity.** First period contributions between Mug and Ice-cream are statistically identical among female co-operators (N = 12 for Mug; N = 15 for Ice-cream. z = 1.001, P = 0.317); within Mug, however, significant differences emerge between female co-operators' (mean = 14.0, N = 12) and male co-operators' in the first period (mean = 17.4, N = 29, z = −2.356, P = 0.019). Nevertheless, over time in Mug, female co-operators' (N = 7 groups) contributions increase so that overall average contributions do not differ between male (N = 12 groups) and female co-operators (N = 7 groups) (z = −0.466, P = 0.641). Table 2 provides evidence to support female conditional cooperation. We see that the coefficient for female conditional cooperation in Mug is 0.635 (z = 5.72, P = 0.000), which is significantly higher than 0.324, the coefficient for male conditional cooperation in Mug (chi2 (1) = 6.94, P = 0.008).
(DOCX)

Table S3 **Allocation of Approval Points.**
(DOCX)

Text S1 (DOCX)

Text S2 (DOCX)

Text S3 (DOCX)

Picture S1 (PDF)
