# Supplementary figures and images for: Competition for Trophies Triggers Male Generosity
Source: PLoS One. 2011 Apr 6;6(4):e18050. doi: 10.1371/journal.pone.0018050 (PMC3071811; doi:10.1371/journal.pone.0018050)

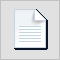

Supplement: Table S1 — Determinants of Approval Received. Table S1 shows that determinants of approval points received follow a similar pattern across the treatments. In particular, the greater (smaller) the contribution in relation to others, the greater (smaller) was the amount of approval a person received. The strength of this effect is identical among treatments. This is shown by the coefficient for “Treatment variable (Baseline/Mug/Ice-cream)×Positive/Negative Deviation from Others' average.” Moreover, in all treatments, the group's highest contributor is also a star winner with frequency at least 90%. (GIF) [file pone.0018050.s001.gif]

## Pictures for the Trophy Mug and the Ice-cream Rewards

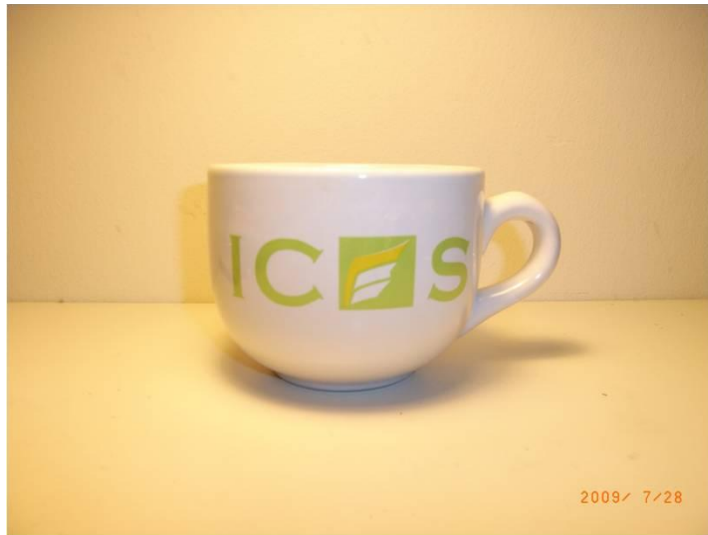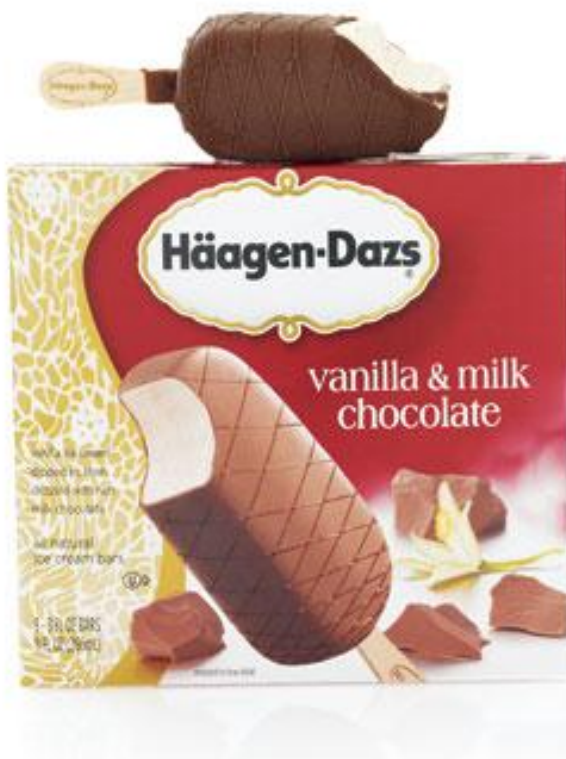

Supplement: Picture S1 — (PDF) [file pone.0018050.s007.pdf]
