# Supplementary material for: Competition for Trophies Triggers Male Generosity
Source: PLoS One. 2011 Apr 6;6(4):e18050. doi: 10.1371/journal.pone.0018050 (PMC3071811; doi:10.1371/journal.pone.0018050)
Supplement: Table S3 — Allocation of Approval Points. (DOCX) [file pone.0018050.s003.docx]

| **Table S3: Allocation of Approval Points** | | |
| --- | --- | --- |
| Random Effect GLS | (1) | (2) |
| Cooperator_Mug | 6.505** |  |
|  | (2.905) |  |
| Cooperator_Mug_Male |  | 6.140* |
|  |  | (3.234) |
| Cooperator_Mug_Female |  | 4.276* |
|  |  | (2.113) |
| Cooperator_Ice-cream | 5.684** | 4.229* |
|  | (2.471) | (2.533) |
| Cooperator_Ice-cream_Male |  | 2.652 |
|  |  | (2.712) |
| Cooperator_Ice-cream_Female |  | 5.954* |
|  |  | (2.970) |
| Free-rider_Mug | 4.908* |  |
|  | (2.731) |  |
| Free-rider_Mug_Male |  | 2.613 |
|  |  | (2.804) |
| Free-rider_Mug_Female |  | 7.997*** |
|  |  | (2.789) |
| Free-rider_Ice-cream_Male |  | -1.633 |
|  |  | (1.598) |
| Pos. Dev. from Others' Avg. Contri in Mug | -.690*** | -.704*** |
|  | (.225) | (.231) |
| Pos. Dev. from Others' Avg. Contri in Ice-cream | -.598* | -.553*** |
|  | (.311) | (.301) |
| Neg. Dev. from Others' Avg. Contri in Mug | -.008 | .024 |
|  | (.225) | (.223) |
| Neg. Dev. from Others' Avg. Contri in Ice-cream | .282 | .313 |
|  | (.191) | (.190) |
| Constant | 7.796*** | 8.782*** |
|  | (1.055) | (1.548) |
| Period Dummies | Yes | Yes |
| # of Obs. | 1040 | 1040 |
| *Note*: Dependent Variable: Approval points assigned by persion *i* in period *t* | | |
| Random GLS regression with robust standard error clustered by group. | | |
