# Supplementary material for: Competition for Trophies Triggers Male Generosity
Source: PLoS One. 2011 Apr 6;6(4):e18050. doi: 10.1371/journal.pone.0018050 (PMC3071811; doi:10.1371/journal.pone.0018050)
Supplement: Text S1 — (DOCX) [file pone.0018050.s004.docx]

**Text S1: Nash Equilibrium in Reward Treatments**

If subjects place sufficient pecuniary value on the rewards, then it is easy to see that positive contributions could be consistent with Nash Equilibrium in reward treatments. Any such pecuniary effects would be identical between reward treatments and thus cannot explain between treatment differences.
