# Supplementary material for: Competition for Trophies Triggers Male Generosity
Source: PLoS One. 2011 Apr 6;6(4):e18050. doi: 10.1371/journal.pone.0018050 (PMC3071811; doi:10.1371/journal.pone.0018050)
Supplement: Text S2 — (DOCX) [file pone.0018050.s005.docx]

**Text S2: Classification of Co-operators and Free-riders**

Each subject is classified as either a Free-Rider or Co-operator. To do this, we first define a decision as cooperative if the contribution for the current period is at least as great as the mean (rounded down to the nearest integer) of the subject’s group members’ contributions from the previous period. A subject is classified as a co-operator if the majority of her nine classifiable decisions (from periods 2 to10) are cooperative; otherwise, she is classified as a Free-Rider.
