# Supplementary material for: Competition for Trophies Triggers Male Generosity
Source: PLoS One. 2011 Apr 6;6(4):e18050. doi: 10.1371/journal.pone.0018050 (PMC3071811; doi:10.1371/journal.pone.0018050)
Supplement: Text S3 — (DOCX) [file pone.0018050.s006.docx]

**Text S3: Controlling for Group Effects**

To control for group effects, each type in each group is associated with a single observation, as follows. Overall, this aims to reduce the enhanced statistical significance caused by inflated number of observations.

For the Approval Points: Each observation is calculated as the overall average of approval points assigned by each type in each group. For example, suppose a group has two female free-riders and two male co-operators. Rather than having two observations for female free-riders, we only have one observation that equals to the average approval points assigned by the two female free-riders. Therefore, in this example we have two observations for a four person group.
