# Supplementary material for: Competition for Trophies Triggers Male Generosity
Source: PLoS One. 2011 Apr 6;6(4):e18050. doi: 10.1371/journal.pone.0018050 (PMC3071811; doi:10.1371/journal.pone.0018050)
Supplement: Supplementary file 6 [file pone.0018050.s002.docx]

Table S2: Dynamic Contribution

	Random Effect GLS	Random Effect Tobit
Mug_male	5.657***	14.479***
	(2.049)	(5.376)
Mug_Female	1.369	6.931
	(2.215)	(6.301)
Ice-cream_Male	1.175	6.245
	(2.157)	(4.977)
Ice-cream_Female	0.778	7.420
	(1.659)	(5.065)
Baseline_Male	.943	3.208
	(1.261)	(4.105)
Approval Points Received	.201***	.286***
	(.030)	(.050)
Male in Mug x Others Avg. Contribution	.324***	.819***
	(.088)	(.200)
Female in Mug x Others Avg. Contribution	.635***	1.277***
	(.111)	(.288)
Male in Ice-cream x Others Avg. Contribution	.339***	.492***
	(.113)	(.224)
Female in Ice-cream x Others Avg. Contribution	.490***	0.793***
	(.085)	(.239)
Male in Baseline x Others Avg. Contribution	.502***	.991***
	(.092)	(.174)
Female in Baseline x Others Avg. Contribution	.580***	1.284***
	(.061)	(.225)
Constant	4.203***	-1.340
	(1.235)	(3.859)
Period Dummies	Yes	Yes
# of Obs.	1368	1368

Note: Dependent variable: Contribution of i in period t, independent variable in period t-1.
Random-effects GLS regression, robust standard error clustered by group
Level of significance: *p<0.1, **p<0.05, ***p<0.01
